# Unveiling the Potential of Functional Components in Hull-Less Barley Grains: Health Benefits, Structural Composition, and Genetic Advancements

**DOI:** 10.3390/foods15050861

**Published:** 2026-03-04

**Authors:** Rizwan Ali Kumbhar, Sadaf Memon, Muzamil Hussain, Yajie Liu, Zongyun Feng, Hui Zhao

**Affiliations:** 1 College of Agronomy, Sichuan Agricultural University, Chengdu 611130, China; rizwankunbhar@gmail.com (R.A.K.); liuyajie@sicau.edu.cn (Y.L.); 2 Agriculture Extension Department, Government of Sindh, Hyderabad 77110, Pakistan; muzamilhussain37@gmail.com; 3 State Key Laboratory of Crop Gene Exploration and Utilization in Southwest China, Sichuan Agricultural University, Chengdu 611130, China

**Keywords:** dietary fibers, protein content, starch content, barley malt, genetic insights

## Abstract

Hull-less barley (HB) has gained attention for its various health supplements and use in beer brewing across China. The role of HB is somewhat limited, accounting for only 2% of the total production for human food; while approximately 6% is used in malt production, HB contains multiple key ingredients and functional components beneficial for health. These include dietary fiber (DF), protein, starch, and barley malt. These components are instrumental in promoting health benefits, including cardiovascular protection, glycemic regulation, lowering blood cholesterol levels, reducing the risk of colorectal cancer, improving cellular signaling, producing short-chain fatty acids (SCFAs), and promoting the growth of beneficial gut microflora. The structural characteristics of HB, such as size and shape, which are important in influencing these traits, are briefly discussed. Additionally, genetics insights into these traits are vital for understanding the molecular mechanisms and gene expression in response to environmental factors. By leveraging genetic studies, we explore the biosynthesis pathways and quantitative trait loci (QTL) regions that influence these health-promoting traits. Given its versatility, HB has the potential to improve cardiovascular health, supporting nutritional food goals, and enhance malting quality. This review highlights HB’s nutritional profile and genetic potential, showing its promise in supporting both health goals and the malting industry.

## 1. Introduction

Cereals are the most widely cultivated crops globally, renowned for their diverse productive and quality uses. Among these, barley (*Hordeum vulgaris* L.) is considered one of these important cereals, cultivated for various purposes, including animal feed, the brewing industry, human consumption, and medical science. Barley is recognized for its functional properties which enrich our daily diet, and ranks as the fourth most cultivated cereal crop worldwide, following wheat, rice and maize, accounting for 12% of the overall cereal production [1]. Typically grown at higher latitudes and in drier regions compared to other cereals like wheat and oats [2], barley is further categorized seasonally into spring and winter types, phenotypically into two-rowed and six-rowed varieties, and by the presence or absence of a hull that firmly adheres to the grain, which classifies it as hulled or hull-less. Additionally, it is differentiated by its end-use type into malting and feeding [3].

Hull-less barley (HB: *Hordeum vulgare* L. var. *nudum* Hook. f.), also known as naked barley, is a self-pollinated annual species characterized by loose husks that detach easily upon threshing and falls off during harvesting, unlike hulled barley [4,5,6]. That distinguishes it from hulled barley, where the husk is firmly attached. It is cultivated globally, with a particular prevalence in East Asian countries such as China, Korea, and Japan. It is also given favor in high-altitude regions, including Tibet, as well as the northern regions of Nepal, India, and Pakistan [7]. The Fertile Crescent in the Near East is renowned for being a principal hub for the origin, diversity, and domestication of barley, as reported by Dai et al. (2012) [8]. Naked barley has been widely cultivated for millennia on the Qinghai-Tibet Plateau of China, where it accounts for 95% of the total cultivated barley, as reported by [9,10]. Spanning Tibet, Qinghai, Gansu, Sichuan and Yunnan provinces, it is the principal grain of the Tibetan people [5]. Additionally, Tibetan barley accounts for over 70% of the total food production in the Tibetan Plateau, as reported by Wu et al. (2025) [11].

On a global level, barley production continues to rise. According to the USDA’s December 2025 Feed Outlook, global barley production for the 2025/26 marketing year is forecasted to reach 152.94 million tons, reflecting a 3.13 million tons increase compared to previous estimates. This increase partially offsets expectations for a reduced global harvest of corn [12]. Major barley producers, including the EU, Canada, Australia, and Russia, have seen production forecasts raised, with the EU’s output increasing by 1 million tons, to 56 million tons [12].

Naked barley requires minimal cleaning in contrast to hulled barley, as there is no need for processing to remove the inedible outer hull. In the United States, most of the barley that has a hull, due to breeding efforts, has focused on malt quality. This is because the hull can offer some benefits [13]. However, HB has received more attention and is valued more highly than hulled barley. This is because the absence of the hull increases the nutrient content of barley grains, which include higher levels of starch, protein, and *β*-glucan [14,15,16]. The genotypes of HB are recognized as offering exceptionally high-quality raw material for nutrition. They have unique and balanced nutrient profiles, with high contents of protein, starch, and non-starch polysaccharides [3,17,18]. It has been noted that six-rowed barley contains a higher protein content compared to two-rowed barley [19], and HB naturally contains a greater amount of protein content compared to hulled barley. In this study, HB was found to have a protein content of 14.23%, while hulled barley recorded 12.35% [20]. Prior to this study, it was reported that barley grains have a protein content that varies between 5% and 18%, with an average of approximately 12% [21].

Barley has a multitude of uses, leveraging its healthy and functional components in a variety of applications, including breakfast items, breads, baby foods, and beverages, to improve their nutritional value. The utilization of barley extends to a direct feed for livestock and poultry, and it is served in food products after pearling, along with its use in brewing and distillation processes to produce alcoholic beverages, followed by malting. Barley is primarily used in the making of alcoholic beverages, such as beer. One valid reason for its limited consumption in the food industry on the large scale is the presence of a husk, which is difficult to remove [22]. Carbohydrates like the cellulose found in spent grains and the *β*-glucan gums that may be by-products of barley processing hold meaningful potential for non-food industrial applications [23].

**Figure 1:**
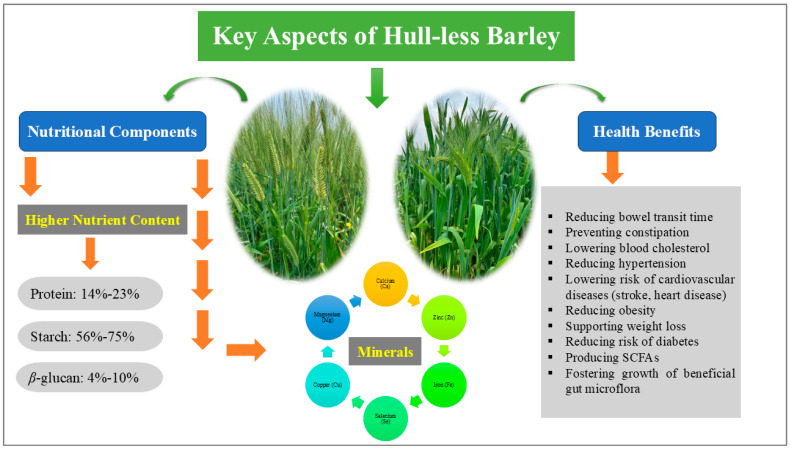
Hull-less barley: nutrient composition and health advantageous overview. This figure illustrates the nutritional components of hull-less barley, including protein (14–23%), starch (56–75%), and *β*-glucan (4–10%). The key minerals mentioned include calcium (Ca), magnesium (Mg), zinc (Zn), copper (Cu), iron (Fe), and selenium (Se). Health benefits include reducing bowel transit time, preventing constipation, and improving cardiovascular diseases.

Barley is abundant in functional components such as antioxidants, lignans, gamma-aminobutyric acid (GABA), minerals, starch and DFs, specifically *β*-glucan, which are beneficial for human health. DFs, known for their ability to emulsify, thicken, and retain water, are frequently incorporated into the formulation of various foods [24]. Barley is a prominent cereal grain frequently found in bread, beverages, and a variety of cuisines across different cultures [25]. In this context, enhancing the quality of bread with efficient fibers like *β*-glucan and insulin holds central importance for both consumers and the cereal industry [26]. Barley, which is rich in BG, also contains significant nutritional value, including proteins, carbohydrates, lipids, vitamins, and minerals [27]. These minerals include calcium (Ca), zinc (Zn), iron (Fe), selenium (Se), copper (Cu), and magnesium (Mg). They are not only relevant to blood sugar regulation, but can also help in maintaining healthy bones and regulating muscle and nerve functions. This concentration of nutrients may further be beneficial in functional foods for diabetics, particularly with the help of *β*-glucan [28]. The DF components of cereal grains, such as *β*-glucan, cellulose, galactomannan, pectin, psyllium, insulin, and resistant starch, are soluble fibers in water, while other fibers, like cellulose, hemicelluloses (e.g., arabinoxylan and mixed-linkage glucans), chitosan, and lignin, are insoluble in water [29,30,31,32]. The water solubility of polysaccharides is influenced by structural factors, such as the degree of branching and the presence of hydrophilic groups. Soluble fibers like *β*-glucan and pectin tend to have branched or amorphous structures that allow for better interaction with water molecules. In contrast, fibers with more crystalline or tightly packed structures, such as cellulose, are less soluble in water. These structural differences are key to their bioactivity and physiological effects [33,34,35]. Therefore, these valuable nutritional elements can enrich the human diet, and their details are briefly described (Figure 1).

HB contains higher starch, protein, and *β*-glucan contents compared to hulled barley, due to the absence of the fibrous hull present in HB [36,37]. Starch, the most plentful natural reserve polysacchride and essential soruce of stored energy for cereal grains [38], is the core final-product of carbon fixation during photosynthesis. It comprises two main components: amylose and amylopectin [39]. The presence of resistant starch, lunasin polypeptide, unique fatty acids, non-starch polysaccharides, and rich phenolic substances gives HB significance potential for promoting anti-cancer, hypoglycemic, and hypolipidemic features, among others [40]. Thus, these phenolic compounds can contribute to the functional properties of barley grain, as these compounds are potential alternatives for bioactive agents in both pharmaceutical and medicinal sectors to promote human health and cure various diseases [41].

Covered barley is traditionally preferred for malting because of its hull, which protects the acrospires during the malting process and provides natural filtration during mashing [42]. Agu et al. (2009) previously demonstrated that modifying malting conditions for HB can achieve good alcohol yield, with significant improvements in processing characteristics [43]. Optimizing conditions through extended steeping time and lowered germination temperatures can further enhance grain modification, improving malt friability and overall quality [44].

### 1.1. Health-Promoting Benefits of Barley

A number of human health benefits associated with barley have been reported. According to [45], the addition of barley to a healthy diet can be highly beneficial, offering a wide range of health advantages, such as support for bone health, immune system enhancement, increased energy, and improved brain and skin health [45]. However, while barley’s potential health benefits are widely recognized, interpretation of its effectiveness across different studies are inconsistent, and further analysis is required to clarify these discrepancies.

Dietary fibers (DFs) in barley, particularly *β*-glucan, are commonly cited for their role in improving human health. Barley consumption has been associated with several benefits, including reduced bowel transit time, prevention of constipation, decreased risk of colorectal cancer, lowered blood cholesterol levels, production of SCFAs [26,46], reduction in hypertension and obesity [47], promotion of weight loss [48], and support for the growth of beneficial gut microflora. Nevertheless, the magnitude of these effects and their practical significance in diverse populations remain subjects of ongoing debate.

A key recognized component in barley’s health-promoting effects is *β*-glucan, which is especially noted for its cholesterol-lowering properties. In particular, *β*-glucan has been shown to help maintain healthy cholesterol levels and promote the development of beneficial gut microbiota [49,50]. A recent study by [51] recommends the consumption of whole-grain barley, suggesting that it offers additional benefits over refined barley, including improved cholesterol management and greater flavor and variety in meals [51]. While the cholesterol-lowering effects of barley are well-established, the literature shows mixed findings on the extent of these benefits. The EFSA panel (2011) concluded that a daily intake of at least 3 g of barley *β*-glucan is required to significantly reduce LDL-cholesterol levels [52]. A Swedish study noted that barley fiber, particularly from whole grains, can lower cholesterol levels in a manner comparable to oat fiber [53]. However, other studies have suggested that the effects of barley on cholesterol may not be pronounced in certain populations or when barley is consumed in refined forms, suggesting that the benefits may depend on factors such as processing methods, individual health conditions, and dietary habits [49,50]. These variations suggest that further research is required to determine the optimal forms and amounts of barley, to maximize its cholesterol level.

Moreover, some studies emphasize the potential role of barley grass in promoting health. For instance, Zhu (2018) highlighted the fact that barley grass can promote sleep, regulate blood pressure, and improve immune function and gastrointestinal health [54]. Additionally, barley grass has been shown to decrease hyperuricemia, prevent hypoxia, reduce fatigue, and alleviate constipation [55]. However, it is important to distinguish between the effects of barley grain and grass, as the latter is often marketed for various health benefits. This distinction requires further research, to clarify the individual contributions of barley grass versus barley grain to overall health.

Barley grains contain anti-oxidant properties that support the body’s fight against cancer by naturalizing free radicals, which can cause DNA damage [56]. These antioxidant qualities may also help to manage blood sugar levels [27]. Modern grain-milling techniques can remove the bran from the grains, where beneficial components like antioxidants and phytoestrogens are concentrated. Consequently, whole grains are more nutritious than refined grain products, due to the presence of bran [57].

Among the most common phenolic compounds found in whole-grain cereals are phenolic acids and flavonoids [58]. The multifaceted mixture of bioactive components in whole-grain foods may offer supplementary health benefits compared to individual, separated components [59]. A recent study demonstrated that these compounds have more health benefits, including improved cellular signaling and critical intestinal defense from undigested polyphenols allied with fibers [60]. However, it is essential to note that the bioavailability of these compounds can vary, with processed barley potentially offering fewer benefits than whole-grain barley.

Additionally, barley amino acids are essential nutrients that play crucial roles in several biological processes, including muscle repair, immunological system function, neurotransmitter generation, and detoxification [61]. A study by Casiraghi et al. (2006) emphasizes the significant influence of barley fiber; when incorporated into various food products, it can reduce postprandial metabolic responses [62]. The consumption of barley has also been linked to a lowering of the risk of cardiovascular diseases, including stroke and heart disease, and to reducing the risk of diabetes [63].

Finally, consumption of barley has been shown to significantly improve the particle-size distribution of high-density lipoprotein (HDL) cholesterol, which is associated with a decreased risk of total stroke [64]. Given these health benefits, it is essential to include regular consumption of barely in the daily diet, in appropriate amounts, to maximize its positive effects and lower the risk of diseases. However, as the literature represents mixed findings, it is crucial to further explore the factors that influence barley’s health-promoting effects and resolve existing contradictions.

### 1.2. Purpose of the Review: Objectives and Scope

The use of HB cultivars has been increasing, highlighting the need to develop winter HB varieties for both domestic and international markets [65]. Although HB currently accounts for only 2–3% of total global barley production, with approximately 6% used in malt production, its potential lies in reducing barley’s antinutrient levels, thereby enhancing the crop’s value by improving its health benefits for humans. This review focuses on the functional properties and ingredients of HB, particularly its role in human health and industrial applications.

HB grains are essential in shaping the nutritional profile of the crop, which has gained increasing attention in countries such as China. This interest centers on its use in the production of economically significant products, including beer, health supplements, and other foodstuffs. However, the molecular studies and genetic mechanisms underlying the traits of interest in HB remain unclear and challenging. Nevertheless, contemporary scientists are actively pursuing research in this area, as the applications of HB are underutilized—particularly in two essential sectors: feed and malt in the food industry.

This review explores the health-promoting components of HB, such as *β*-glucan, proteins, and starch; malt quality; the role of DFs in industrial applications; and the structural composition of barley grains. We also examine the genetic developments associated with barley. Specially, China has shown growing interest in exploring the functional traits of HB grains, including *β*-glucan, protein, starch, and malt potential. This review discusses the potential applications of these traits and the ongoing genetic studies aimed at optimizing HB’s uses in various sectors.

## 2. Functional Components of Barley

Barley is well-known for its various health and nutritional benefits. The most significant functional components of HB are discussed below:

### 2.1. Dietary Fibers

DF consists of indigestible parts of plant cells, which are resistant to hydrolysis by human enzymes [35]. DFs can be divided into soluble and insoluble types, based on their solubility and ability to form viscous solutions. Soluble fibers dissolve in water, while insoluble fibers do not. The soluble components of DFs are particularly crucial for influencing their physiochemical properties and physiological functions [66].

A recent study demonstrated that whole barley contains around 32.2 g of total dietary fiber per 100 g dry matter, which includes both soluble and insoluble dietary fibers, explaining its high fiber content compared to many cereals [67]. In contrast, another study showed the average range of total fiber in barley varies from 15.0–23.8%, depending on variety and processing, with soluble fiber varying accordingly [68].

Physiologically, the framework of DF comprises five constituents: backbone structure, water-holding capacity, structural charge, fiber matrix, and fermentation rate [69]. These components can influence the health-promoting properties of fibers. Additionally, fiber is considered a type of functional food. It is part of an expanding list of examples of diet–microbe–host interactions that connect microbial, metabolic and immune host responses [70]. While some studies have demonstrated that fiber intake can improve gut microbiota composition and immune function, other studies report fewer clear results. Below, we briefly discuss *β*-glucan and barley *β*-glucan, along with their limited applications in food.

#### *β*-Glucan

**Figure 2:**
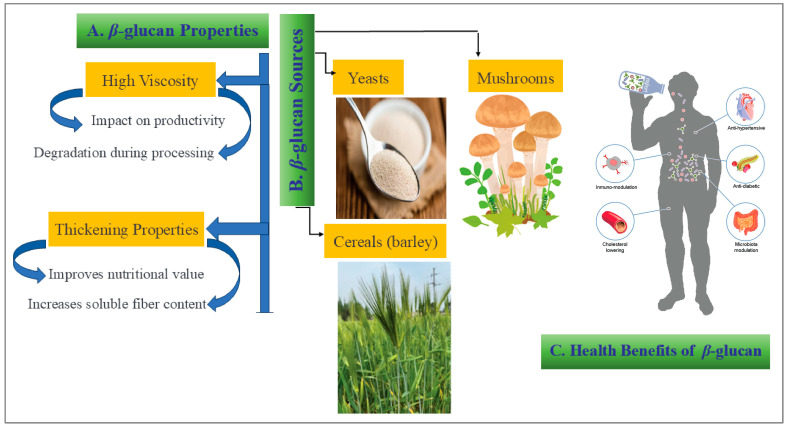
Section A describes the high viscosity and thickening properties of *β*-glucan, which influence the productivity and degradation during processing. These properties also enhance the nutritional value and soluble fiber content of food products. Section B identifies the primary sources of *β*-glucan (yeasts, mushrooms, and barley). The final section, C, illustrates the multiple health benefits associated with *β*-glucan consumption, such as its anti-hypertensive, anti-diabetic, cholesterol-lowering, and microbiota-modulating effects, as well as its role in immune modulation.

*β*-glucan has unique properties, and potential health benefits have attracted significant attention in recent years. Numerous previous studies have demonstrated that *β*-glucan increases the viscosity of intestinal contents in chicks consuming barley, which consequently leads to a reduction in productivity [71]. This high viscosity is a characteristic feature of *β*-glucan (depicted in Figure 2), which is regularly degraded during extraction and subsequent processing stages [72].

Among hydrocolloids, *β*-glucan is notable for its thickening properties, which can improve the nutritional value of various food products by increasing their soluble fiber content [1]. The level of *β*-glucan widens with maturity, which indicates a lower solubility of the *β*-glucans [73]. Various sources of fibers and polysaccharides such as mushrooms, yeasts, and cereals, contain *β*-glucan, which has been documented to possess antitumor, anti-microbial, anti-allergic, and immune-modulating properties [74].

While the presence of *β*-glucan is likely to alter the degradation of starch, there has been less consideration given to *β*-glucan content in terms of degradation kinetics [75]. Notably, during bread making, *β*-glucan can be partially degraded by endogenous enzymes, affecting its molecular weight and functionality in the final product. Among cereals, barley is recognized for its higher *β*-glucan content, further highlighting its nutritional importance.

### 2.2. Barley β-Glucan

Barley and oats are recognized for their comparatively high level of *β*-glucan, a bioactive component that plays a beneficial role in human health when consumed as part of the diet. *β*-glucan has been widely recognized for its health-promoting properties, and those have already been discussed in the section above. However, it also poses an undesirable influence on the malting process, raising concerns in both agricultural and food-science contexts. Additionally, the industrial and food applications of *β*-glucan, as well as the potential use of HB in beer brewing, have been previously discussed in our review [76].

**Figure 3:**
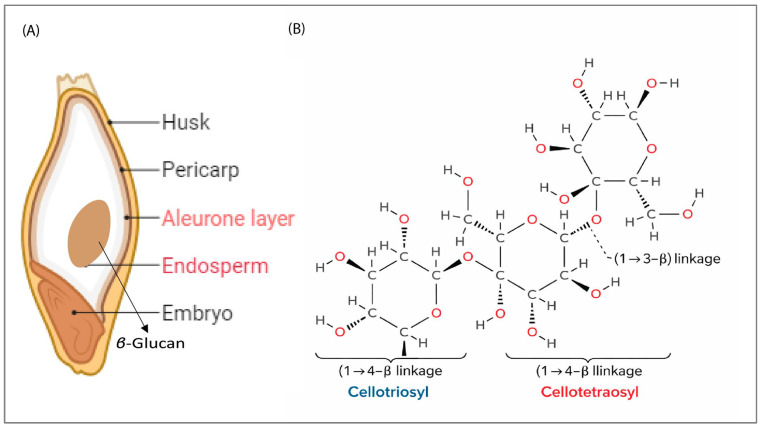
(**A**) Cross-section of a barley seed showing the endosperm and aleurone layer, where *β*-glucan is concentrated. (**B**) Chemical structure of *β*-glucan with labeled cellotriosyl and celletraosyl units, and (1→4)-*β* and (1→3)-*β* linkages. The blue colour highlights the cellotriosyl units, the red colour represents the celletraosyl units, and the black colour indicates the *β*-glucan linkages.

*β*-glucan is primarily located in the sub-aleurone layer of the cell walls within the endosperms of barley and oats [77,78,79]. HB barley contains 4% to 10% *β*-glucan, which is higher compared to other cereals [80,81]. Another earlier study also supports this finding, reporting 4% to 9% *β*-glucan in barley [82]. This makes HB specifically valuable for producing functional barley flour, which can be easily incorporated into multiple foods to meet the authorized health claims associated with *β*-glucan [83]. Research indicates that *β*-glucans are non-starchy, non-digestible polysaccharides found in the walls of barley endosperm cells. These polysaccharides are predominantly formed by cellotriosyl and cellotetraosyl units that are linked by single (1→3)—*β*-linkages [84] (Figure 3A,B). However, contradictory findings exist regarding the solubility of *β*-glucan in different barley varieties. For instance, Abdel-Gawad et al. (2016) reported that, regarding *β*-glucan solubility, it remained higher in flours from hulled barley compared to HB [85]. The presence of these mixed linkages regulates the final physical properties of the *β*-glucan, including solubility and viscosity [86]. Moreover, the contribution of (1→3, 1→4) *β*-linkages to the overall solubility and viscosity of *β*-glucan is debated.

For malting barley, a lower *β*-glucan level is typically preferred, as high *β*-glucan content is thought to reduce malt quality by increasing viscosity during brewing. In China, research has identified high BG content which has been investigated as a key factor that reduces the malting quality of local barley varieties [87]. Thus, to achieve higher quality cereal grains, particularly those with elevated *β*-glucan contents, it is essential to consider various factors. Different strategies may be necessary for the distinct crops and varieties involved [47]. Furthermore, the *β*-glucan content in barley can vary not only among different varieties, but also due to environmental conditions. This variation is closely linked to genetic factors that influence the type of starch and hull present in the grains [88].

### 2.3. Barley Protein

The protein content of barley is of considerable importance, due to its significant contribution to the nutritional value of barley-based products, including bakery product items and animal feed. Barley is considered a cost-effective source of food proteins, due to its substantial protein content [89]. Protein content is a key factor in determining the quality of barley for breeding purposes, as it improves the nutritional and commercial value of the crop [90,91]. Barley proteins account for approximately 8–27% of the total grain weight and perform various functions, including storage, structural roles, and metabolic activities in commercial applications [91]. However, while most studies report that grain protein content (GPC) of barley typically ranges from 8% to 12% [92,93], early studies by Raj et al. (2023) suggest a broad variability, with value ranging between 10% and 20%, particularly concentrated in the endosperm [27]. This discrepancy highlights the heterogeneity of protein content in barley, which is well-documented to be influenced by genetics and growing conditions.

For the malting and brewing industries, barley grains with protein content ranging between 9.5% and 11.5% of dry weight are considered optimal, especially when more than 90% of the harvested grains (such as the grain-retention fraction) are larger than 2.5 mm [94]. Barley hordeins constitute 50% or more of GPC in mature seeds, which is important for flour quality, as demonstrated by [95]. The proteins present in barley grains play crucial roles in structural functions, facilitating metabolic processes, and supplying nitrogen necessary for embryo growth.

These proteins in barley are generally categorized into two main groups: seed storage proteins (SSPs) and structural proteins. Non-storage proteins are predominantly located in the aleurone and embryo regions, while storage proteins are mainly found in the endosperm [96]. The major storage proteins in barley belong to the prolamin group, primarily composed of hordeins, which accounts for approximately 30% to 50% of the barley protein fraction [97]. Prolamins, which are the major type of protein in barley, are alcohol-soluble proteins that play a critical role in nutrient storage in barley seeds. Other storage proteins in barley include albumins (soluble in water), globulins (soluble in salt solutions), and glutelins (soluble in diluted acid or alkali solutions) [98,99,100,101,102]. Storage proteins accumulate primarily in protein storage vacuoles (PSVs) and in protein bodies (PBs), which are derived from the endoplasmic reticulum (ER) [103]. These proteins are essential not only for germinating seedlings, but also for key sources of nitrogen and sulfur, which are important for crop development and as dietary proteins for humans and livestock [104].

Various published studies have demonstrated that agrochemical methods, along with environmental and cultural field practices, can influence the amount of protein in barley. Nitrogen fertilization is a well-established agronomic method to manipulate barley GPC, with significant implications for grain quality and end uses [105]. However, excessive nitrogen application can negatively impact on malting quality and malt extract. Additionally, the rate and timing of nitrogen application also influence protein accumulation and associated enzyme activities (e.g., *β*-amylase) [106]. This suggests that careful management is required to maintain desired quality traits for specific end uses of barley.

The relationship between protein content and barley grain quality is complex and can vary, depending on the intended use of the barley. For feed applications, high protein content is generally desirable because it improves the nutritional quality of the feed. However, for malting purposes, lower protein levels are typically preferred [96], because high protein can interfere with starch degradation, affecting the quality of the malt, wort, and beer [107,108]. Additionally, Marquez-Cedillo et al. (2000) provide a review indicating that GPC is a preliminary commercial requirement for the malting trait in barley and is associated with various of the other quality traits [109].

### 2.4. Presence of Starch in Hull-Less Barley

**Figure 4:**
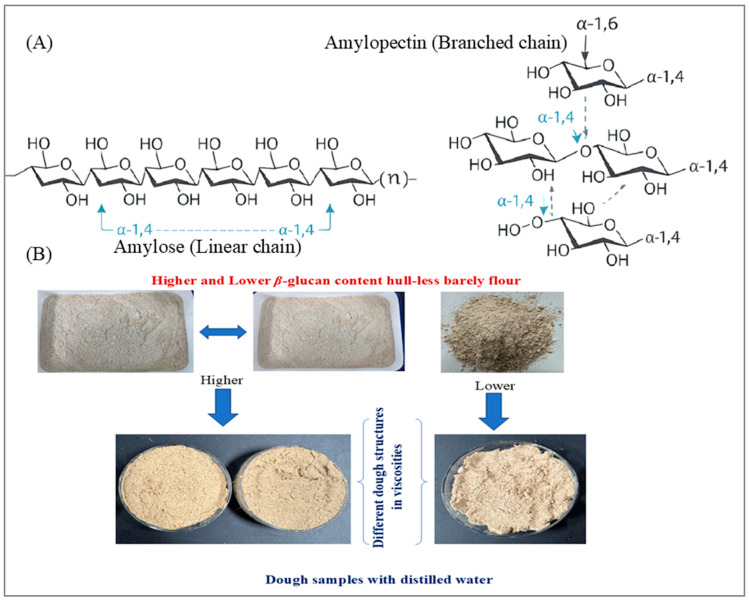
(**A**) Chemical structure of starch (amylose and amylopectin). (**B**) Dough structure and viscosity of hull-less barley flour with higher and lower *β*-glucan content.

Starch is a key functional component in HB, providing several benefits for a healthy diet and lifestyle. Over the years, research has shifted from traditional uses to more advanced studies exploring the functional properties of barley starch in the context of dietary fiber and resistant starch (RS). The grain-filling process of HB involves starch accumulation [39], playing a critical role in its functional properties. Starch is composed of glucose molecules that are linked together to form complex carbohydrate polymers. These polymers occur in two main forms: amylose (a linear polymer) and amylopectin (a branched polymer) (Figure 4A) [110]. Amylose makes up approximately 5–35% of natural starches and significantly impacts the functional properties of starch in food [111], such as its water absorption and texture.

Amylose, along with lipids, phosphorylated deposits, and long lateral-chain amylopectin, interacts to limit water uptake, influencing starch texture and functionality in food [112]. However, the proportion of amylose and amylopectin can vary with barley variety, affecting the RS content and the health benefits of barley-based products. The estimation of amylose and amylopectin content in starch can be achieved through iodine staining or by combining this technique with other fractionation methods, providing a detailed analysis of starch composition [113]. Moreover, studies have reported that the ratio of amylose to amylopectin impacts the properties of starch-based products.

Starch is the predominant component in barley kernels, accounting for approximately 56 to 75% of the dry kernel weight. However, this percentage can vary, depending on the barley variety [114,115]. Other studies report that starch is the largest component in barley grains, making up about 65% of the kernel dry weight [116]. This variation highlights the need for standardization of methods to assess starch content across different barley varieties and environmental conditions.

RS is a type of starch that is not digested by enzymes in the small intestine, but instead passes into the colon, where it is fermented by gut microflora [117]. The incorporation of RS in cereal foods such as bread, cookies, biscuits, and pasta increases the fiber content and induces modifications in in the product’s texture, color, and sensory properties [118]. For example, Devi et al. (2024) demonstrated that dough’s rheological properties significantly impact critical aspects of bread production, including workability and shape, and the development of a well-structured crumb and crust [119]. These properties also allow for adjustments during mixing, fermentation, and baking, ensuring the consistent production of bread with desirable qualities such as texture, volume, elasticity, and mouthfeel [119].

Viscosities in barley can be affected by both developmental factors and barley varieties, with the most substantial influence stemming from compositional transformations, particularly the starch and *β*-glucan content [120,121,122]. Figure 4B illustrates the differences in dough structure and viscosity outcomes when HB flour with varying BG and starch contents used. The central and bottom images depict dough samples prepared with distilled water, highlighting the distinct dough structures and viscosities. Significant viscosity breakdown was observed during flour dough preparation, indicating that the rheological properties of the dough are significantly impacted by starch composition.

The incorporation of RS in barley-based foods can improve their health benefits, including glycemic control, regulation of fasting plasma triglyceride and cholesterol level, improved mineral absorption, prevention of elevated blood sugar, and reduced risks associated with cancers and cardiovascular diseases [123,124].

RS exists in several forms, each explaining important characteristics regarding digestion and absorption. These types include RS2, RS3, RS4, and RS5. RS1 is non-accessible and physically entrapped within the cellular matrix, such as in partially or whole milled grains [125]. RS2 consists of ungelatinized resistant granules found in high-amylose corn starch, green bananas and raw potatoes [126]. RS3 is retrograded starch, which forms in cooked and cooled foods like potatoes and bread; it has a slower digestion, and is reversable [117]. RS4 refers to the fact that it has been chemically modified to resist enzymatic digestion through the addition of starch ethers and cross-linking with chemical reagents [117,127,128,129].

**Figure 5:**
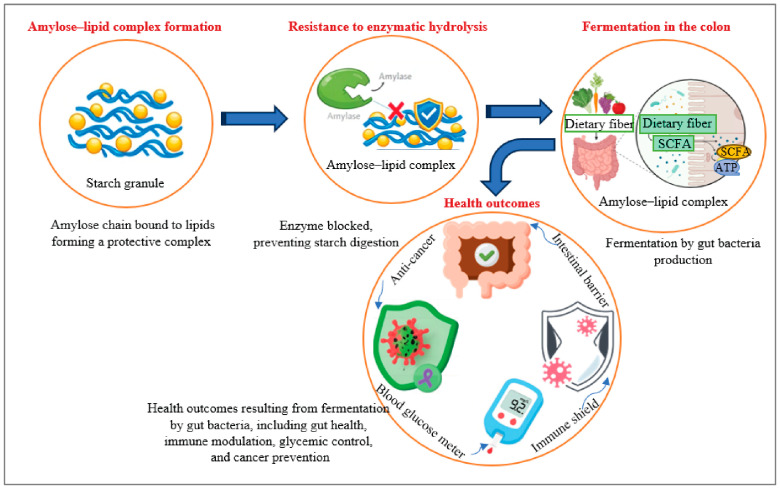
Schematic representation of amylose–lipid complex formation, resistance to hydrolysis, and health outcomes. The “x” symbol indicates enzyme blockage, preventing starch digestion, while the “√” symbol represents enzyme activity, allowing starch digestion. Health outcomes resulting from fermentation by gut bacteria, including gut health, immune modulation, glycemic control, and cancer prevention.

The final category, RS type 5, is composed of amylose–lipid complex, which exhibits resistance to enzymatic hydrolysis [130,131]. In this complex, amylose chains (long chains of glucose molecules) bind to lipid molecules within the starch granule, preventing enzymes, such as amylase, from acting on the starch. This binding inhibits starch expansion and leads to a structural change in the starch granule, which impedes enzymatic breakdown. Consequently, the amylase–lipid complex behaves similarly to DFs, and, upon reaching the colon, is fermented by gut bacteria into SCFAs (Figure 5), which are metabolites produced by intestinal bacteria. These SCFAs play several crucial roles in gut health, including preserving the integrity of the intestinal barrier and modulating immune function [132].

Other significant health benefits of RS5 are its ability to control postprandial glycemic and insulinemic responses, as well as its potential in the prevention of colon cancer [133]. Studies have found that lipids bind to amylose within the starch granule, inhibiting its expansion and thus conferring resistance to enzymatic hydrolysis. These amylose–lipid complexes are typically formed from high-amylose starches, such as those found in corn and other specific cereal crops [134]. Thus, each of these RS types has unique properties that may influence their digestibility and health benefits, ranging from gut health to metabolic regulation.

### 2.5. Barley Malt

Barley malt is primarily used in the production beverages such as beer and whiskey, making it a key ingredient in the brewing industry [135]. For malt production, achieving a varietal purity of 95% is crucial [136]. Malting refers to the process of formulating barley for brewing purposes through partial germination, which is induced by drying. This process softens the barley-grain cell walls and promotes the growth of diastatic enzymes. These enzymes covert starch into malt extract [137]. Several enzymes containing β-glucanase are synthesized, and their activities decompose storage materials such as protein, starch, and *β*-glucan [138]. The capability of barley grain to produce maximum levels of (1→3, 1→4)-β-glucanase, specifically, appears to be an essential indicator of malting quality [139]. Careful attention can be paid to malting conditions during production to address issues associated with *β*-glucan. Barley malt, along with hops and malt, are considered as rich sources of polyphenols, and minerals including calcium, iron, magnesium, phosphorous, potassium, zinc, and selenium, as well as vitamins, all of which are incorporated into beer [130,140]. Studies have also found that moderate beer consumption can have positive effects on cardiovascular health and metabolism [130].

#### The Conversion of Barley into Malt

The conversion of barley into malt is a multi-stage process involving several carefully controlled steps. According to Narwal et al. (2020), the procedure of barley malting may involve three discrete stages: steeping (also known as soaking), germination, and kilning [141]. During the conventional steeping, the grains are soaked in water to increase the level of moisture by approximately 42–47% [142]. During germination, heat and carbon dioxide are produced as a result of the metabolic activities within the grains. The steeping stage itself has specific characteristics. It consists of alternating periods where the grains are immersed in water with dry periods. While the steeping process lasts between 36 and 48 h, some studies suggest that adjusting the steeping time based on barley variety and environmental conditions may enhance moisture absorption and protein content [143,144].

**Figure 6:**
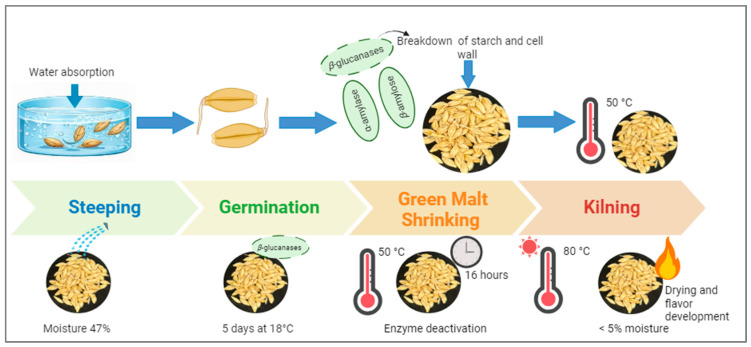
Biochemical and biological pathways during barley malting: a schematic overview of the barley malting process from steeping to kilning, illustrating the enzymatic breakdown of starch and cell walls, temperature regulation, and moisture-content changes at each stage.

Although germination is usually carried out at 18 °C for 3 to 6 days [145], some studies indicate that higher temperatures or shorter periods may be used to optimize enzymatic activity, particularly for brewing purposes. The second step is to continue the germination of steeped barley to acquire “green malt” (Figure 6). This green malt is categorized through high moisture contents of approximately 47% and maximum enzymatic activity, resulting in the hydrolysis of cell walls and starch mediated by α-amylase, *β*-amylose, and *β*-glucanases [146]. In this step, germination enables the embryo to develop under controlled conditions. During this process, the seed develops enzymes that lead to the breakdown or modification of endosperm structure. Furthermore, enzymes hydrolyze and dissolve the stored starch and protein of the endosperm [147]. Taking this malting parameter, in the current study, the barley grain was germinated for 5 days at 18 °C. The green malt was then dried at 50 °C for 16 h, followed by kilning at 80 °C for 4 h, to reduce the water content to less than 5% [148].

Kilning is the final step in the malting process, where heat treatment dries the green malt and avoids further germination. During this process, the moisture content of malt is decreased below 5%, which ensures the solubility of the product for storage purposes and transport, and to stop the denaturing of enzymes. Kilning further promotes the development of melanoidins through the nonenzymatic Maillard reaction between amino acids and sugars [137,149]. Furthermore, the drying process further reduces moisture content and facilitates flavor development and browning reactions, which contribute to the malt’s characteristic color.

## 3. Structural Composition of Barley Grain

Barley grain, an indehiscent fruit known as a caryopsis, consists of several key components that contribute to its nutritional and functional properties in food applications. These caryopses emerge from spikelets, which are allied to the rachis of the barley spike by short structures known as rachillas. Classified by their elongated shape, barley grains feature a longitudinal crease that runs the whole length of the grain, effectively dividing it into two halves [150]. The three primary components of barley are the embryo (germ), the outer layers (including the husk, pericarp, testa, and aleurone layer), and the endosperm [135]. Together, these components are crucial for the grain’s nutritional composition, contributing significant amounts of fiber, *β*-glucan, starch, and proteins, which are essential for promoting health benefits in barley-based products.

During grain development, the hull—composed of cellulose, lignin, and silicon—forms the outermost layer, which is adhered in hulled barley, but detached in naked barley [96]. This trait makes naked barley particularly useful in the food industry, where it is processed into barley flour for baking or incorporated into functional food products. The structural composition of the barley grain includes a large endosperm, containing 80% of the barley grain [151]. The grain typically measures 7–8 in length, and has a spindle-shaped form [152]. Overall, the composition of these grains influences cereal digestion in both human and animal guts, significantly contributing to the nutritional value and potential health benefits of barley-based products [153].

**Figure 7:**
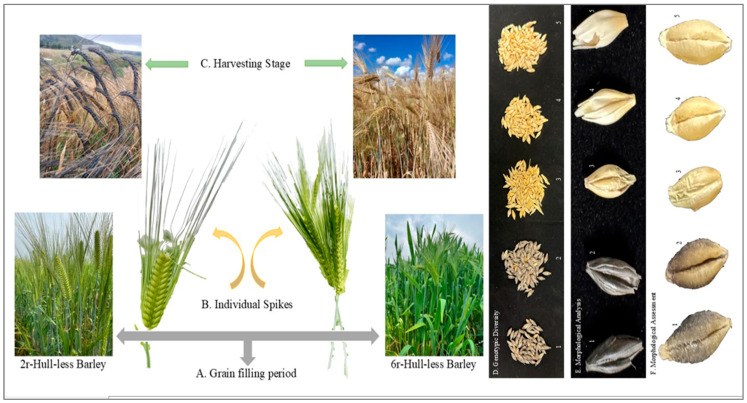
Developmental stages and genotypic color variation in two-rowed and six-rowed hull-less barley. Panel A represents the grain-filling period in HB, while panel B shows individual spikes of two-rowed HB, with awns longer than those of six-rowed HB. Panel C depicts the maturation stage, showing the growth of HB as it progresses toward ripening, with changes in grain colour and texture. Panel D illustrates color variation among five different genotypes. Panel E displays individual seeds of the same varieties, along with their detached hulls. Panel F shows hull-removed grains with a similar appearance.

Although it is generally observed that smaller grains contain higher protein levels and lower starch content, some studies report that environmental factors (such as temperature, water stress, and nutrient availability) can influence this relationship, causing larger grains to also exhibit higher protein content in certain cases [135]. For example, under drought stress, larger grains may accumulate more protein than smaller grains. Barely grains are commonly divided into two color types: a bright light-yellow and off-white. Unwanted discoloration can negatively affect malting quality [3]. In one of our recent field studies, black grains were observed in two-rowed HB, which is common (illustrated in Figure 7).

The variation in color among barley cultivars and landraces is linked to the presence of flavonoid pigments in the seed’s pericarp and aleurone layer [154]. Since barley grains play a key role in various applications, the important components of the grain are described as follows:

### 3.1. Caryopsis

The caryopsis (covered or naked barley) is an important agronomic trait, particularly in terms of dietary use and food processing [155]. Hulled caryopses have hulls—comprising adaxial (palea) and abaxial (lemma) layers—which firmly adhere to the pericarp on maturity, while naked caryopsis lack these adhering hulls [156]. The adhesion between the husk and caryopsis is influenced by the composition of the cementing layer, which is regulated by temperature conditions during grain development [157]. For industrial purposes, naked barley is more suitable for direct consumption and processing into barley flour, which is used in baking and functional foods because of its hull-free nature. The barley husk, while less useful in food directly, has potential applications in biodegradable packaging, biosensors, and food-grade adsorbents [158]. Additionally, the shape of the caryopsis—whether straight or twisted—distinguishes two-rowed and six-rowed barley, which influences grain processing for malting and brewing [159]. The presence of hulls in hulled barley is beneficial for animal feed and brewing purposes, as the hull and pericarp layers are strongly adhered at maturity, contributing to malt quality [155].

### 3.2. Embryo

The embryo is the most vital part of the barley grain, serving as the origin for the next generation of crop plants. It results from the reunion of male and female reproductive cells, and is the principal living organ within the grain, encircled by the aleurone layers [135]. Barley seeds do not exhibit a specific period of dominancy, and the aleurone cells are consistent in size and shape across species [25,160].

Additionally, the barley embryo offers essential nutrients for the growth and development of plants [161]. The endosperm provides the energy required for seed germination, and its protein and starch content significantly influence its suitability for feed and food processing [161]

### 3.3. Endosperm

The endosperm of barley, which is softer and more flexible than that of wheat [162], is the basic storage tissue within the grain. The endosperm is composed mainly of *β* glucan, arabinoxylan, and cellulose, which are significant in food products for their functional properties. *β*-glucan, which accounts for 75% of the cell wall in the endosperm, is a major dietary fiber with health benefits such as lowered cholesterol and improved gut health [163]. The endosperm also stores fats, protein, and starch, supporting the growth of the embryo and serving as a key ingredient in malting [25]. Furthermore, the endosperm’s architecture governs the uniform distribution of moisture and enzymes, which is essential for consistent modification during malting [164], a crucial step in brewing and the production of malted beverages. The deposition of *β*-glucan occurs during the later stages of grain filling, indicating that conditions favoring endosperm development can enhance *β*-glucan accumulation [87]

### 3.4. Variations in Grain Size and Shape

Grain size and shape strongly affect barley processing and the quality of derived foods. Larger grains generally result in higher malt yields and improved seedling vigor, which benefits both the malting industry and the feed industry [165,166]. Additionally, grain size and shape affect the texture and quality of food products, including bread, snacks, and gluten-free items. The range of grain size in barley results from both evolutionary variability and agronomic selection processes, as well as environmental factors and the genetic architecture of barley species [167,168].

While other grain parameters, such as grain weight, grain width, and area, also hold significance, physical characteristics like size, shape, and color, along with nutrient composition, serve as distinctive features among cereal grains [169]. Over time, barley grains have improved in size and shape, possibly due to factors such as environmental adaptation, human selection, and fluctuations in agricultural practices [170]. In HB (naked barley), the tendency of grains to lose their hull before harvest is a key trait that enhances processing efficiency, especially for malting and food milling. The genetic basis of threshability remains poorly understood, but it plays a significant role in barley-grain processing [171].

## 4. Genomics and Molecular Approaches to Enhancing Hull-Less Barley-Grain Quality

Genomics is an advanced approach that is implemented in practice to study how genes function and are regulated in external environments. Currently, climate change has become a global challenge, and HB is vulnerable to environment variations. While both genotypic and environmental factors influence yield-related traits in HB, there is debate about the relative impact of each. Some studies suggest that environmental stress (such as drought) might play a more significant role in shaping yield traits in HB than previously thought. However, genetic factors also have a stronger impact on trait determination, particularly when selection is carried out in controlled conditions. This inconsistency highlights the complexity of genotype x environment interactions, and underscores the importance of both forward genetics and molecular breeding to understand the molecular mechanisms underlying these crop traits.

Barley is diploid in nature and adapted to diverse environments, offering inimitable resources for genetic research and crop improvement [172]. The genetic regulation of barley traits is typical, and the naked caryopsis trait is one example. The trait, which is a domestication-related characteristic, is defined by the loss of function of the *nud* gene. The *nud* gene encodes an Apetala 2/Ethylene-Response Factor (AP2/ERF) that regulates the development of the cementing layer, including the pericarp, lemma, and palea [173]. Previous studies have indicated that the hulled caryopsis in barley is governed by a single locus (*nud*) located on the chromosome arm 7H [174]. However, the specific role of the *nud* gene remains unclear, while the *nud* gene is generally accepted as playing a major role controlling the naked hull phenotype [175]. Several studies have identified the location of the *nud* gene on chromosome 7H, but the exact impact of its mutation on other barley traits, such as recessive to environmental stresses or grain quality, is still not fully understood [176]. For example, Cas9 endonuclease has been used to knock out the *nud* gene in the covered variety ‘Golden Promise,’ creating a naked isogenic line for studying the pleiotropic effects of the gene [42]. 

For a long time, barley has been genetically transformed using a wheat thioredoxin *h* gene (*wtrxh*) driven by a seed-specific promoter, aiming to target the expression of the gene product to the endosperm [177]. Barley caryopsis itself is a rich ingredient, and its quality is categorized by a variety of key traits, including GPC, amylose content, starch content, and malt-quality characteristics such as malt extract, wort viscosity, Kolbach index value, free α-amino nitrogen, and diastatic power [3,178,179,180]. Additionally, genomic prediction has proven effective in supporting the targeted selection of populations with high breeding potential by evaluating hybrid performance across germplasm [181].

### Transcriptomic and Metabolomic Approaches in Barley Research

Transcriptomic sequencing and metabolic analysis are powerful approaches that, when combined, enable researchers to evaluate and investigate genes of interest and their interactions associated with targeted traits in HB. By integrating these two omics approaches, researchers can gain a more comprehensive and holistic understanding of cellular processes and mechanisms underlying desired traits in barley. Recent studies have demonstrated that integrated analysis of metabolites and transcriptomes can reveal the intricate interaction between gene expression and metabolite accumulation, offering valuable insights into underlying regulatory mechanisms [182,183]. These studies outline methods for integrating metabolic and transcriptomic data, offering a framework to explore gene–metabolite interactions across diverse organisms. The adaptation of such approaches could enhance our understanding of metabolic regulation and gene expression in crops, such as barley.

Metabolomics involves the quantitative detection of all metabolites and their biochemical states within specific organisms or tissues [184]. This approach allows for a schematic view of the metabolic landscape in barley, detecting the metabolites responsible for desirable traits such as *β*-glucan, GPC, and starch content. On the other hand, transcriptomics provides real-time information related to gene-expression profiles, which is essential for understanding the genetic basis of complex traits. It is commonly used for identifying functional genes involved in biosynthesis pathways [185].

RNA-seq is an advanced and widely used approach for transcriptome profiling, utilizing deep-sequencing technologies [186]. This method allows for high-throughput analysis of gene expression, and, importantly, it can validate the reference genome, thereby making the results more reliable [155]. For instance, RNA-seq performed on growing and germinating barley seeds from annotated genes in the QTL regions has provided additional validation of the functional roles these genes play in modulating seed *β*-glucan content [187]. This is particularly valuable for identifying genes that influence nutritional traits and for optimizing breeding strategies aimed at improving barley quality.

Additionally, transcriptomics can reveal differentially expressed genes (DEGs) under various environmental conditions, while transcriptomic analysis provides insights into gene expression and observable phenotypic changes. Techniques such as metabolomics, biochemical assays, cytometry, and proteomics also play a role of a bridge between genes and phenotypes [188]. Considering the importance of barley grain, this chapter provides an in-depth overview of the genetic aspects of its nutritious traits, such as *β*-glucan, protein, and starch content, as well as malting quality. However, integrating transcriptomic and metabolomic data remains complex, owing to the inherent differences in data types. We therefore suggest that more refined integration methodologies are needed to fully leverage the potential of these approaches in barley research

## 5. Genetic and Molecular Mechanisms of *β*-Glucan Biosynthesis in Barley

**Figure 8:**
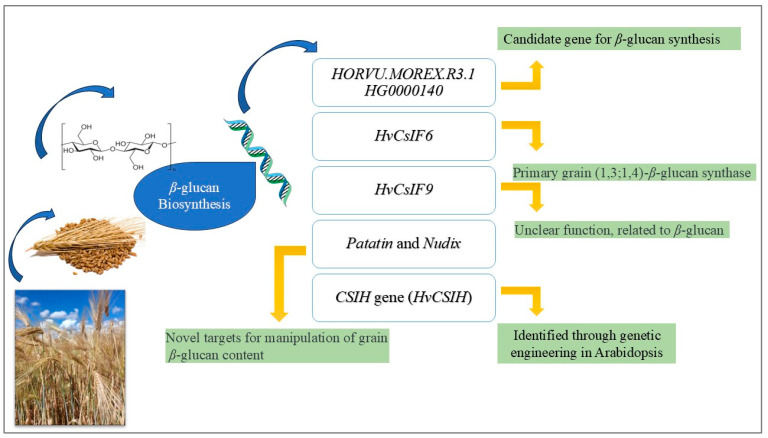
Identified genes involved in (1,3;1.4) *β*-glucan biosynthesis in barley. This figure illustrates the key genes and their functions in the biosynthesis of *β*-glucan in barley. It shows the pathway from barley grains to *β*-glucan synthesis, highlighting candidate genes for genetic manipulation aimed at increasing *β*-glucan content, including *HvCslF6*, *HvCslF9*, the primary grain (1→3, 1→4) synthase, and the *CSIH* gene, as well as genes like *Patatin* and *Nudix*. These genes have roles that are not yet fully understood, but are related to *β*-glucan synthesis.

*β*-glucan is a polygenic trait in HB, significantly influenced by both genetic and environmental factors. Several candidate genes in barley have been identified through annotations in the barley genome [189,190] (Figure 8). However, despite numerous studies, the exact genetic mechanisms underpinning *β*-glucan biosynthesis remain complex and sometimes contradictory. For instance, *β*-glucan is known to be influenced by diverse germplasms and environments, which has led to unpredictable QTL locations [191].

In grains of barley, disruption of the cellulose synthase-like gene *CsIF* eliminates (1,3;1,4)-*β*-glucan, which is advantageous for the distilling and brewery industries. One of the most studied genes in this context is the *CslF6*, which is considered the main isoform contributing to the total mixed-linkage glucan (MLG) in both vegetative and floral tissues of barley [192,193,194].

Qi et al. (2024) demonstrated that the *CSlH* gene (*HvCSIH*) could be genetically engineered into *Arabidopsis* through the transformation method, leading to *β*-glucan biosynthesis in the cell wall [195]. Although this study shows success in transgenic plants, the translation of these findings to barley and other cereals has been slower. The gene expression of *HvCsIF6*, while crucial in grain *β*-glucan synthesis, varies across barley cultivars and environmental conditions. For instance, the *HvCsIF9* is known to be expressed during mid and late grain-development stages, while *HvCsIF9* is primarily active during early growth stages (8–10 DPA) [196]. This suggests that while *HvCsIF6* is a predominant *β*-glucan synthase gene, *HvCsIF9* may also have a role, albeit less clearly defined, in the early stages of barley development.

Furthermore, expression and functionality of other *HvCsIF*-family genes have shown inconsistent results in different studies. Although *HvCsIF6* is regarded as indispensable for *β*-glucan synthesis, the role of *HvCsIF9* remains unclear. Some studies suggest that *HvCsIF9* has complementary role in early grain development, while others indicate its expression is less significant in comparison to *HvCsIF6* [197,198]. These results highlight a gap in our understanding of the complete gene set responsible for *β*-glucan biosynthesis, and emphasize the need for further research to clarify their interactions and contributions.

In addition to the *Csl* genes, other novel targets for *β*-glucan manipulation in barley have been identified. Research has highlighted the potential of *Patatin* and *Nudix* hydrolase as targets for altering grain *β*-glucan content [187]. Recent studies also identified the gene *HORVU.MOREX.R3.1 HG0000140*, located near a significant single nucleotide polymorphism (SNP) locus B1_1033963, as a potential contributor to *β*-glucan synthesis [199]. These results indicate key genomic regions that could be targeted for genetic improvement.

The *HvCsIF* subfamily, consisting of ten members [200], has been a focal point for gene editing strategies like CRISPR/Cas9 to manipulate β-glucan levels in cereal grains. Among these, *HvCsIF6* and *HvCsIF9* are considered prime candidates for targeted gene editing to enhance *β*-glucan content for various applications in human nutrition, livestock feed, and in the malting/brewing industries [201]. Furthermore, evidence has shown that the *CSLH* gene also play a role in *β*-glucan synthesis when expressed in transgenic Arabidopsis [202], yet the extent of this gene’s contribution across different plant species remains inconsistent, suggesting that other genetic factors or environmental conditions may influence its activity. A recent study identified significant SNPs positioned near the *CslF* family on chromosome 7H, highlighting the important regions for genetic improvement [203]. However, the link between these SNPs and actual changes in *β*-glucan content is still unclear, and requires investigation.

Nemeth et al. (2010) identified the *CslF6* gene in wheat as a putative *β*-glucan synthase, demonstrating its function through RNA interference (RNAi) suppression in transgenic wheat [204]. While this finding supports the role of *CslF6* in *β*-glucan synthesis, in recent research, an effective Agrobacterium-mediated transformation system for the HB cultivar Torrens was developped, achieving a transformation efficiency of approximately 1.8%. Using this, *HvCsIF6*, a *β*-glucan synthase gene driven by an endosperm-specific promoter, was over-expressed, resulting in increased MLG levels in HB grains. However, concerns have been raised about the potetnial negative impact of ovcerexpressing *HvCslF6* on cell wall structrue, as HB cultivars appear to be more sensitive to modifications in cell wall composition [205]. Overall, these findings support the hypothesis that *β*-glucan content is regulated by QTLs, and that the expression of cellulase gene family members, particularly *HvCsIF6*, plays a crucial role in *β*-glucan biosynthesis [206].

However, it is important to acknowledge that *β*-glucan regulation is complex, with genetic variation, environmental influences, and the potential off-target effects of gene editing contributing to the observed variability in *β*-glucan content.

## 6. Genetic and Molecular Mechanisms Underlying Protein and Starch Biosynthesis in Barley

The GPC in barley is determined by both environmental and genetic factors [207]. A variety of genes are involved in regulating GPC, as demonstrated by numerous mapping studies [208]. While genome-wide association studies (GWASs) provide greater genetic variation and higher resolution in mapping phenotypes at the population level compared to conventional QTL mapping [209], the results of GWASs for GPC in barley have been somewhat inconsistent. For instance, a study by [93] reported that Tibetan barley exhibited higher GPC than cultivated barley. However, a GWAS in the same study identified two *HvNAM* genes (*HvNAM_1_* and *HvNAM_2_*) as candidate genes for GPC control, but no significant association was found between *HvNAM_1_* polymorphism and GPC. In contrast, a polymorphism in the second intron of *HvNAM_2_* was associated with increased GPC. This discrepancy suggests that the genetic control of GPC may be more complex than originally thought, with environmental factors potentially playing a significant role in gene expression and trait manifestation [93].

Further complicating these findings, an earlier study mapped an orthologous gene chromosome 6H in barley, where a QTL for the GPC trait was identified, and the *NAM_1_* gene was found to exhibit limited allelic variation across wild- and cultivated-barley genotypes [210]. Additionally, a previous study reported that both the *HvNAM-1* and *HvNAM-2* genes are mapped on chromosome number 6H and 2H, respectively [211]. Although the *HvNAM_1_* and *HvNAM_2_* gene was not significantly associated with GPC in some populations, other studies have pointed to its potential as a source of genetic variation for improving grain nutrition in specific barley genotypes, such as Nordic barley [212]. This indicates that *HvNAM* genes are promising candidate genes for GPC enhancement, and that their role may be genotype-independent, underscoring the challenge translating these findings across diverse barley varieties.

Another contradiction arises from studies focusing on G-protein genes in barley [213] identified eight barley G-protein genes, including *HvGα1*, *HvGβ1*, *HvGγ1*, *HvGγ2*, *HvGγ3*, *HvXLG1*, *HvXLG2*, and *HvXLG3*, with uneven chromosomal distribution, primarily across chromosomes 4, 5, 6, and 7. These genes are thought to be involved in regulating barley’s response to environmental stressors, with some studies suggesting their potential role in GPC regulation. However, the actual influence of GPC remains uncertain, with conflicting results regarding their involvement in protein accumulation [213]. For instance, certain QTLs like QGFmt-6H, QGFhd-7H, and QGFhd-2H have been associated with protein content, yet the exact mechanisms through which these genes regulate GPC remain unclear, and likely depend on environmental conditions [214].

Distelfeld et al. (2008) identified a barley QTL for GPC near the marker *hvm74* on chromosome 6H, which is potentially orthologous to wheat’s *Gpc-B1* gene [215]. This gene encodes the NAC transcription factor *TtNAM-B1*, which is associated with increased grain protein, zinc, and iron content [215]. Both the *HvNAM-1* and *HvNAM-2* genes are mapped on chromosome number 6H and 2H, respectively [211]. Various previous studies have also reported findings that define similar QTL regions on certain chromosomes. Furthermore, modifications in mapping populations, molecular markers, and experimental environments across these studies contribute to variations in QTL results [216]. Consequently, it remains challenging to precisely determine QTL locations and identify candidate genes associated with barley quality-related traits [180]. In previous studies, six environmentally stable QTLs for GPC were identified, located on chromosomes 2H, 4H, 6H, and 7H. Notably, three QTLs on chromosome 7H, designated as QGpc.ZiSc-7H, QGpc.ZiSc-7H.2, and QGpc.ZiSc-7H.3, were identified in couple phase, marking the first report of such linkage among these loci for GPC in this barley species [217].

Starch is synthesized in the endosperm of growing barley grains, serving as the primary reservoir for stored carbohydrates [218]. Starch synthases (SSs) play a crucial role in incorporating glucose units into starch, with several enzyme classes involved in starch biosynthesis. Among these, SSs facilitate the elongation of both amylose and amylopectin chains [219,220]. A recent study reported newly identified barley mutants, *hvbe2a-1* and *hvbe2a-2*, which developed extended starch granules (SGs) in the endosperm, absent in the wild type. These mutants had genetic lesions in the *HvBE2a* gene, which codes for a major branching enzyme (BE) in barley endosperm [221]. However, while the role of SSs in starch biosynthesis is widely accepted, some studies suggest that the contribution of certain starch synthase isoforms may be tissue-specific, with distinct roles in the endosperm compared with other grain tissues. This consistency highlights the complexity of starch biosynthesis and the need for further studies to clarify these differences across barley tissues.

The study identified key genes involved in starch biosynthesis during the development of HB grain. Notably, genes encoding sucrose synthase (*Hvulgare_GLEAN_10012370*, *Hvulgare_GLEAN_10021199*), ADP-glucose pyrophosphorylase (*Hvulgare_GLEAN_10033640*, *Hvulgare_GLEAN_10056301*), and starch-branching enzymes 2b (SBE2b; *Hvulgare_GLEAN_10018352*) were significantly expressed during grain maturation in both Zangqing 2000 (Q) and 08-1127 (C2) [222]. These discoveries align with previous studies on the importance of these enzymes in starch accumulation. However, discrepancy findings exist in the literature regarding the tissue-specific expression of some of these genes. The profile of starch accumulation aligns with the expression of key starch biosynthetic genes, with fewer genes active compared to the endosperm. Notably, only *SS2b* and *SS1* are expressed in the pericarp, suggesting that starch structure may differ from endosperm-storage starch [223].

Ref. [131] conducted a study on 100 barley accessions, which were divided into two groups based on the polymorphism of the marker S5H_29297679. Ninety-three accessions possessed the GG allele, while seven accessions had the AA allele. This study identified four putative candidate genes: *HORVU6Hr1G087920*, *HORVU5Hr1G011230*, *HORVU5HrG011270*, and *HORVU5Hr1G011280*, which were found to be highly expressed during barley grain development, specifically during rapid starch accumulation [131]. These findings appear promising, but the study’s conclusions on the role of these candidate genes are not entirely conclusive, especially considering the variation in gene expression across different barley genotypes and growing conditions. The study also found that the SNP markers for starch content were concentrated on chromosomes 1H and 4H, corresponding to loci *qSC1-1* and *qSC4-1*, respectively [224].

However, ref. [225] conducted a larger GWAS on 658 barley accessions, focusing on grain-quality traits including GPC, grain starch content (GSC), extractivity (EX), and grain test weight per liter (TWL). This study identified the relative significant associations for QTL_Q12 (ipbb_hv_6) and QTL-Q29 (ipbb_hv_128), which influenced GPC, GSC, and EX, providing valuable insights for marker-assisted selection in barley breeding [225]. In contrast, Borém et al. (1999) reported QTLs influencing starch granule traits, which were detected in two supplementary regions: one located on chromosome 4H and the other on chromosome 5H [226]. These results differ from those of Zhang et al. (2020), who focused their findings on 1H and 4H [227]. This variability implies that starch granule synthesis is controlled by multiple, genotype-specific QTLs, whose detection depends on barley germplasm and experimental design. Based on the information provided in this chapter, Table 1 summarizes the genes and proteins involved in important traits related to GPC, starch biosynthesis, and starch granule traits in barley. It lists the key genes associated with each trait, along with their corresponding QTL regions and relevant references.

## 7. QTL Hotspot Regions and Genes Associated with Barley Malting Quality

**Figure 9:**
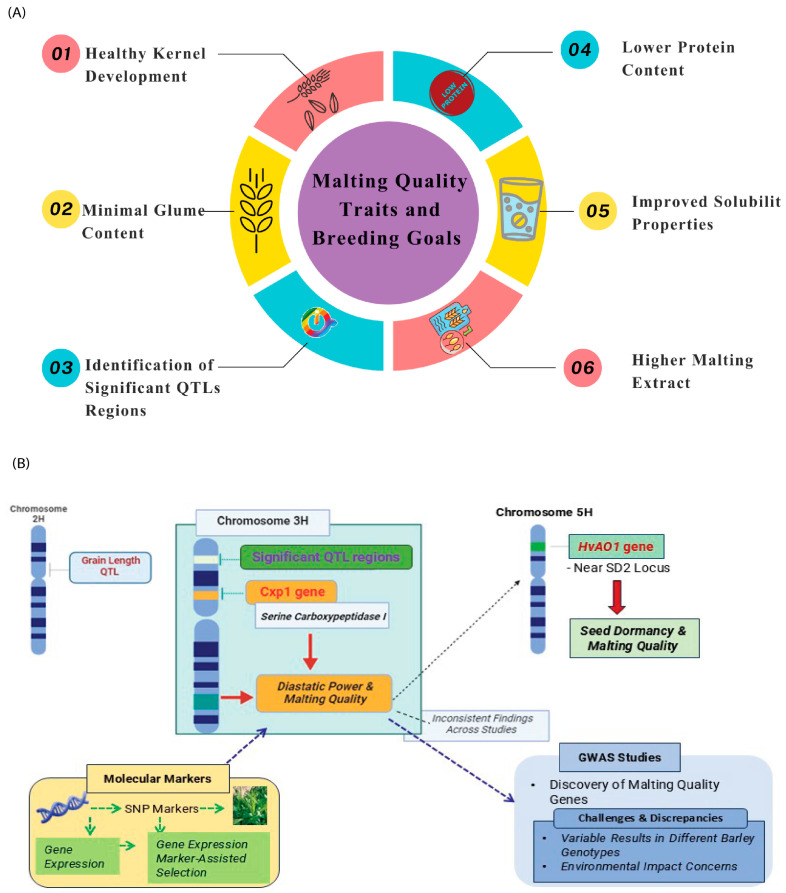
Barley malting-quality improvement diagram and malt extract and solubility: genes and QTL regions in barley. (**A**) This diagram illustrates the key traits and breeding goals associated with malting quality in barley, including healthy kernel development, minimum glume content, and higher malting extract. (**B**) This panel emphasizes the genes and QTL regions identified in barley associated with malting quality. It shows the relationship between chromosome regions such as those on 2H, 3H, and 5H, and their influence on traits like malt extract, solubility properties, and seed dormancy.

The definition of malting is dependent on brewing processes. Generally, the main breeding goals for malting barley include higher malting extract, lower protein content, improved solubility properties, healthy kernel development, and minimal glume content [229]. In this context, Figure 9A illustrates a diagram that centrally positions these objectives and explains how they are interconnected, emphasizing the multifaceted nature of malting-quality improvement.

The present study revealed that QTL mapping identified several novel loci controlling malting quality, which could be further exploited in marker-assisted selection. In this context, a complex QTL region on the short arm of chromosome 3H is of particular interest, as it harbors several loci with significant effects on malting traits [230]. However, discrepancy outcomes from other studies suggest that this QTL region on chromosome 3H may not be universally consistent across different barley genotypes. Moreover, a recent study employed nine molecular markers to evaluate malting quality among barley varieties and breeding lines. These markers demonstrated an 81% probability of reliably predicting malt quality [231]. Meanwhile, there is another argument about reliability of molecular markers across diverse genetic backgrounds.

In this study, two main grain-length QTL were identified at positions parallel to the QTL for malt extract on chromosome 2H and the *uzu* gene on chromosome 3H. However, the grain-length QTL on 2H was more likely to be distinct from the malt-extract QTL, as important candidate genes are located outside the fine-mapped QTL region for malt extract [232]. This distinction between the grain-length and mal-extract QTL is further supported by other studies that propose different genes controlling these traits.

Recent research has highlighted the barley aldehyde oxidase 1 (*HvAO1*) gene as a key factor linked with seed dormancy and malting quality. In a study by [233], the *HvAO1* gene is situated near the SD2 (seed dormancy 2) locus at the telomeric end of chromosome 5H (illustrated in Figure 9B). These results suggest that *HvAO1* variants could be leveraged to minimize SD and barley-malting quality through targeted breeding strategies [233]. Meanwhile, molecular markers from barley SNP arrays that target a given gene (associated with a trait of interest) and provide a connection to the gene’s expression profile [234,235] are gaining a grip in barley breeding schemes. The specificity and accuracy of these SNP markers, particularly for multi-allelic genes, could lead to variable expression, depending on the genetic background of the barley varieties. A decade ago, Potokina et al. (2004) identified several candidate genes associated with barley-malting quality, including serine carboxypeptidase I (*Cxp1*) [236]. The same gene was mapped as an SNP marker on chromosome 3H using a Steptoe (feeding grade) x Morex (malting grade) mapping population. Only a single QTL was identified that significantly influenced the expression level of the *Cxp1*. This expressed QTL maps to a region on chromosome 3H that corresponds to the structural gene and is associated with a QTL for “diastatic powder,” one of the many traits measured to evaluate malting quality [237]. These findings may further highlight the need for refinement of the genetic mapping of malting traits to clarify the relative contribution of different genes.

Moreover, following QTL identification, various GWASs have also been effectively conducted on important agronomic traits associated with grain quality, including protein content and malting-related features in barley [238,239]. Indeed, GWAS has led to the identification of important genes associated with malting quality. However, some studies have failed to replicate the findings of previous GWASs, raising questions about the transferability of identified markers across different genetic backgrounds and environmental conditions. This discrepancy suggests that while GWAS provides a powerful approach for gene discovery, its application in applied breeding may require further validation and integration with other breeding strategies.

## 8. Conclusions

Several studies have highlighted the key importance of HB, a variety extensively grown in Tibetan areas, which contains grains with a range of important nutritional traits. This review focused on the role of dietary fibers (specifically *β*-glucan content), protein content, starch, and barley malt. Increasing the levels of *β*-glucan, proteins, and starch in HB is a primary breeding goal, driven by consumers’ demand for healthier diets. In contrast, for malting barley, which is used in alcohol production (e.g., beer), lower protein and *β*-glucan content are often preferred. Studies have shown that moderate beer consumption can have significant positive effects on cardiovascular health and metabolism, further underlying the value of HB in both health and industry.

Each of these barley components holds substantial value not only in agricultural and food sciences, but also in medical sciences due to their potential influence on human health, including lowering blood cholesterol, aiding weight loss, and reducing risk of diabetes. For example, barely has long been used in traditional medicine in forms such as talbina (a porridge made from barley flour, milk and honey) to promote a healthy heart [240]. These functional components play a crucial role in dietary supplements and functional food applications.

The quality of barley grains must be carefully evaluated before fermentation, as lower grain-malt quality can negatively impact the quality of the resulting products, including alcohol. Genetically, this review incorporates molecular insights into QTL regions, molecular mechanisms, and genes associated with these traits. Advances in barley pangenomics and functional genomics have opened new frontiers in barley research, offering valuable insights to assist scientists and breeders in decision-making for future pre-breeding efforts [235,241,242].

In light of this, it is crucial to deepen our understanding of the genetic mechanisms underlying key quality traits of barley, such as β-glucan, protein, and starch content, as well as malting quality. Given the growing commercial demand for healthier and more sustainable food products, enhancing the utilization efficiency of these traits in HB through molecular breeding may provide fruitful results for future breeding programs.

## 9. Future Perspectives

1. Functional Food Development: the future of HB bioactives lies in the development of functional foods and dietary supplements. Bioactive compounds, such as *β*-glucan and antioxidants, have demonstrated potential in reducing the risk of chronic diseases, like type 2 diabetes, cardiovascular disease, and obesity. Future research should focus on enhancing the bioactive potential of HB through targeted genetic breeding programs. Specifically, efforts should aim at increasing *β*-glucan content, improving antioxidant levels, and exploring barley’s potential in diverse functional food products, such as barley-based snacks and beverages that combine health benefits with consumer convenience.
2. Health-Promoting Properties: it has been well noted that phenolic compounds in barley, in combination with *β*-glucan, offer enhanced health benefits, including improved cellular signaling and enhanced intestinal defense. Future studies should focus on how the complex mixture of these bioactive compounds in whole-grain barley can offer synergistic health benefits compared to isolated components. Additionally, studies should investigate the mechanisms underlying these synergistic effects and their implications for barley-based food products in promoting human health.
3. Quality Assessment: there is a need for standardized methods to assess the quality of both HB grains and malt. Additional research should aim at developing reliable quality-control protocols that can be applied across the food and brewing industries. Additionally, studies should investigate how the quality of barley grains and malt influences the end products, including alcoholic beverages and foodstuffs, and how to develop policies to mitigate undesirable effects during processing.
4. Enhancing Barley Protein Quality and Stability: barley protein is renowned for its high amylose and lysine content. Expression of both hordeins and glutelin genes ensures nitrogen-use efficiency, and directly influences the functional and nutritional properties of barley grains. Various strategies can also be employed to enhance ethe stability and bioavailability of prolamins (e.g., hollow nanoparticles and glycosylation). Future research should focus on optimizing sustainable agronomic practices to improve protein content in HB, while minimizing the negative impacts of excessive nitrogen fertilization. Innovations in genetic engineering approaches, such as gene-editing techniques and RNA interference (RNAi), could be crucial for enhancing protein quality by silencing specific genes that affect prolamin stability and bioavailability.
5. Barley Starch Optimization for Health and Functional Foods: the ratio of amylose to amylopectin in barley starch plays a key role in the formation of RS, which has significant health benefits, such as glycemic control and gut health improvement. Future studies should focus on optimizing the amylose/amylopectin ratio in barley through breeding or genetic modification. Additionally, research should explore how the incorporation of RS into barley-based foods can enhance their texture and sensory properties, providing new opportunities for functional food development.
6. Forward Genetics: understanding the genetic mechanisms underlying the biosynthesis of traits related to functionality, such as *β*-glucan, protein content, and starch composition, is essential for optimizing barley-malt quality. Future research should utilize forward genetics and genomic tools, such as CRISPR/Cas9, to identify key genes and QTL associated with these desirable traits. By manipulating these genes, researchers can enhance barley’s functional properties and improve its suitability for diverse food applications.
7. Breeding Schemes and Goals: the development of HB varieties should align with breeding objectives that focus on optimizing levels of DFs, protein, and starch to meet the needs of both health-conscious consumers and the brewing industry. Molecular breeding approaches, such as marker-assisted selection and gene editing, should be employed to increase these traits while decreasing anti-nutritional factors. Finally, research should also focus on the role of HB in sustainable diets, with particular consideration given to its potential as a source of plant-based protein and DF for addressing global food-security challenges and climate resilience.

## Figures and Tables

**Table 1:** Barley protein and starch biosynthesis: Genes and QTLs.

Trait	Gene/Protein Involved	QTL Regions	References
GPC	*HvNAM1*, *HvNAM2*, *HvGα1*, *HvGβ1*, *HvGγ1*, *HvGγ2*, *HvGγ3*, *HvXLG1*, *HvXLG2*, *HvXLG3*	Chromosome 6H (nud locus), Chromosome 7H (QGpc.ZiSc-7H)	[93,208,212]
Starch Biosynthesis	Starch Synthetases (SSs), *HvBE2a*, Sucrose Synthase, ADP-Glucose Pyrophosphorylase, *SBE2b*	Chromosomes 1H, 4H (qSC1-1, qSC4-1), Chromosome 3H (*uzh gene*)	[218,221,222,228]
Starch Granule Traits	NAC transcription factor *TtNAM-B1* (associated with *Gpc-B1*)	Chromosomes 4H and 5H (QTL_Q12, QTL-Q29)	[215,225,226]

## Data Availability

No new data were created or analyzed in this study. Data sharing is not applicable to this article.

## Abbreviations

*β*-glucan	(1→3, 1→4)-beta-glucan
HB	Hull-less barley
GWAS	Genome-wide association study
SNP	Single-nucleotide polymorphism
QTL	Quantitative trait loci
*Csl*	*cellulose synthase-like*
DF	Dietary fiber
SSPs	Seed-storage proteins
RS	Resistant starch
RNA-seq	Ribonucleic acid sequence
MLG	Mixed-linkage glucan
GPC	Grain-protein content
SSs	Starch synthases
SBE2b	Starch-branching enzymes 2b
SD	Seed dormancy
SCFAs	Short-chain fatty acids
